# Biomimetic Nanosystem Loading Aggregation‐Induced Emission Luminogens and SO_2_ Prodrug for Inhibiting Insufficient Photothermal Therapy‐Induced Breast Cancer Recurrence and Metastasis

**DOI:** 10.1002/advs.202405575

**Published:** 2024-07-21

**Authors:** Ni Zhang, Wei Ping, Meng Suo, Zeyuan Zhang, Wenhai Zhang, Tianfu Zhang, Shipeng Ning, Ben Zhong Tang

**Affiliations:** ^1^ Department of Thoracic Surgery Tongji Hospital Tongji Medical College Huazhong University of Science and Technology Wuhan Hubei 430030 China; ^2^ School of Biomedical Engineering Affiliated Cancer Hospital & Institute of Guangzhou Medical University Guangzhou Medical University Guangzhou 511436 China; ^3^ Department of Breast Surgery The Second Affiliated Hospital of Guangxi Medical University Nanning 530000 China; ^4^ School of Science and Engineering Shenzhen Institute of Aggregate Science and Technology The Chinese University of Hong Kong Shenzhen (CUHK‐Shenzhen) Guangdong 518172 China

**Keywords:** aggregation‐induced emission, cancer stem cell, gas therapy, insufficient photothermal therapy, tumor recurrence and metastasis

## Abstract

Photothermal therapy (PTT) holds considerable clinical promise. However, insufficient PTT‐induced tumor recurrence and metastasis is an urgent practical problem that needs to be solved. Herein, a biomimetic mesoporous organosilicon nano‐system called PSAB is designed to precisely deplete cancer stem cells (CSCs) and prevent tumor recurrence and metastasis after PTT. The PSAB system is made up of Aggregation‐induced emission (AIE)‐active photothermal agent, 2TT‐*o*C26B, and SO_2_ prodrug, benzothiazole sulfinate (BTS), within mesoporous organosilicon nanoparticles (MON) enclosed by an exterior platelet membrane. PSAB effectively targets CSCs both in vitro and in vivo by P‐selectin/CD44 interaction. The degradation of MON and subsequent release of BTS and AIE molecules are facilitated by intracellular glutathione (GSH). Subsequently, the acidic tumor environment triggers the SO_2_ gas therapy from BTS. This process leads to the depletion of GSH and CSCs elimination. After combining PSAB with photothermal therapy, there is no significant tumor recurrence or metastasis. These results indicate that SO_2_ gas therapy and AIE‐mediated PTT act synergistically to offer a unique approach for preventing tumor recurrence and metastasis after PTT, thus holding significant promise for clinical applications in cancer PTT.

## Graphical abstract

Insufficient Photothermal therapy (PTT)‐induced tumor recurrence and metastasis is an urgent practical problem. Herein, the PSAB nano‐system is designed to precisely deplete cancer stem cells and prevent tumor recurrence and metastasis after PTT. When PSAB is used in combination with PTT, there is no significant tumor recurrence or metastasis. These results offer a unique approach for preventing tumor recurrence and metastasis after PTT.

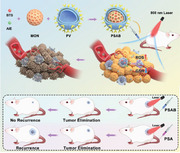

## 1 Introduction

Breast cancer has emerged as the primary cause of cancer‐related mortality among women, with a rising global incidence rate.^[^ ^1^ ^]^ While various treatment approaches such as radiotherapy (RT) and chemotherapy have been developed for the critical scenario,^[^ ^2^ ^]^ their limited response rates and unavoidable adverse effects have hindered the practical application. In recent years, nanomaterials and nanotechnology have been widely studied in tumor treatment.^[^ ^3^ ^]^ There has been significant interest in photothermal therapy (PTT) that could generate thermal energy upon near‐infrared (NIR) laser excitation of photo‐absorbing compounds, resulting in the thermal ablation of tumor cells.^[^ ^3^, ^4^ ^]^ The NIR‐laser exhibits enhanced tissue penetrating capabilities and minimal absorption by biological tissues, rendering it advantageous in cancer therapies.^[^ ^3^, ^5^ ^]^ Organic photothermal agents (OPTAs), such as porphyrin and cyanine dyes, have garnered significant interest because of their favorable attributes, such as low toxicity, excellent biocompatibility, and structure tunable.^[^ ^6^ ^]^ Nevertheless, traditional OPTAs face common limitations, including poor water solubility and susceptibility to photobleaching. These OPTAs exhibited large conjugation and planar conformation, which tend to form π–π staking in the aggregate state and hinder their intramolecular motion, resulting in unsatisfactory heat conversion efficiency in the physiological environment.^[^ ^6^, ^7^ ^]^ To address these challenges, researchers have made significant progress in developing a novel type of OPTAs known as aggregation‐induced emission (AIE) luminogens (AIEgens).^[^ ^8^ ^]^ With the highly twisted conjugated structures, stacking can be avoided in the aggregate state and afford AIEgens adjustable molecular motions.^[^ ^9^ ^]^ Thus, these unique materials possess exceptional resistance to photodegradation and remarkable heat conversion efficiency when aggregated in an aqueous environment,^[^ ^10^ ^]^ emerging as promising OPTA candidates for cancer therapy.

Nevertheless, despite these advancements, PTT has shown limited efficacy in completely eradicating tumor cells, particularly cancer stem cells (CSCs), leading to tumor recurrence.^[^ ^11^ ^]^ CSCs, which are characterized by their ability to self‐renew, differentiate, and proliferate indefinitely, are widely recognized as a significant contributor to tumor metastasis and recurrence following RT, CT, and surgical treatment.^[^ ^12^ ^]^ In contrast to non‐stem cancer cells, clinical and experimental research has yielded significant information indicating that CSCs can withstand PTT through many mechanisms.^[^ ^11^ ^]^ These mechanisms include the adjustment of cellular heat tolerance, upregulation of reducing chemicals such as glutathione (GSH) to facilitate cell repair, and modification of their division patterns.^[^ ^10^ ^]^ Their inherent stemness confers resistance to hyperthermia and ultimately allows them to evade the cytotoxic consequences of treatment. Hence, it is highly desirable to develop a novel therapeutic approach to overcome CSCs resistance and resolve the considerable clinical obstacles posed by metastasis and recurrence after PTT.

Gaseous molecule‐based gas therapy is an innovative approach to support synergistic therapy with other cancer treatments.^[^ ^13^ ^]^ Numerous gaseous molecules, including hydrogen sulfide, hydrogen, and carbon monoxide (CO), have demonstrated the ability to sensitize cancer cells.^[^ ^14^ ^]^ Sulfur dioxide (SO_2_), a gas molecule, has a long history of being recognized as a conventional air pollutant.^[^ ^15^ ^]^ In recent years, several studies have documented the significant contributions of SO_2_ in combating various diseases. Despite its toxicity when inhaled, SO_2_’s extreme oxidative stress can be used to enhance intracellular reactive oxide species (ROS) and trigger death in cancer cells by depleting GSH.^[^ ^15b^ ^]^ The significance of SO_2_ in cancer therapy lies in its oxidative capabilities, which are expected to play a crucial role in treating CSCs. Unfortunately, the majority of documented therapeutic methods, including gas therapy, have mainly focused on treating subcutaneous tumor models, neglecting to address the issue of cancer recurrence caused by the presence of CSCs. Recurrence of the tumor is a common occurrence even after the entire tumor has been surgically removed.

**Scheme 1:**
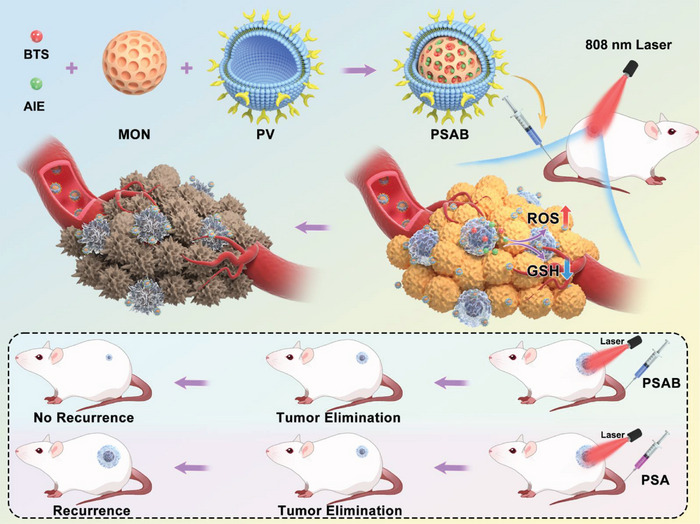
Schematic illustration of biomimetic nanosystem loading aggregation‐induced emission luminogens and SO_2_ prodrug for inhibiting tumor recurrence and metastasis after photothermal therapy.

In this work, a GSH/NIR‐initiated biomimetic nanosystem with the combination of AIEgen‐mediated PTT and SO_2_ gas therapy is constructed to selectively target and eliminate CSCs, hence preventing tumor recurrence and metastasis after PTT. In detail, the nanosystem defined as SAB was formed preliminarily by co‐loading an AIE photothermal agent, 2TT‐*o*C26B, and the SO_2_ prodrug, benzothiazole sulfinate (BTS), within disulfide‐bridged organic mesoporous silicon (MON). Subsequently, platelet‐derived vesicles were applied to coat the surface of the SAB, synthesizing the biomimetic PSAB (**Scheme** **1**). The presence of the P‐selectin protein on the platelet membrane surface, with a strong affinity for the CD44 receptor highly expressed by tumor cells, facilitated the endocytosis process of PSAB and resulted in an improved capacity for prolonged blood circulation and enhanced tumor targeting. After accumulating in the cancer cell, the overexpressed GSH could break the disulfide bond in MON to initiate the release of the loaded AIEgen and BTS for amplified PTT and SO_2_ gas therapy. Hence, GSH levels in CSCs were reversely reduced by SO_2_ production in the acidic tumor microenvironment, which triggered the enormous formation of hydroxyl radicals and ultimately caused CSCs death. The in vivo finding indicated that tumor recurrence was inhibited in mice receiving PSAB with laser irradiation but not in those of other control groups, which could be attributed to the CSCs ablation power of synergistic SO_2_ gas therapy and AIEgen‐mediated PTT by PSAB. This study intended to demonstrate the advancement of a combinational therapy system that effectively eliminates CSCs with a strong emphasis on biosafety and promising prospects for clinical application. The nano‐system reported in this investigation is considered an innovative therapeutic approach that demonstrates effectiveness in preventing insufficient PTT‐induced tumor recurrence and metastasis.

## 2 Results and Discussion

### 2.1 Preparation and Characterization of PSAB

**Figure 1:**
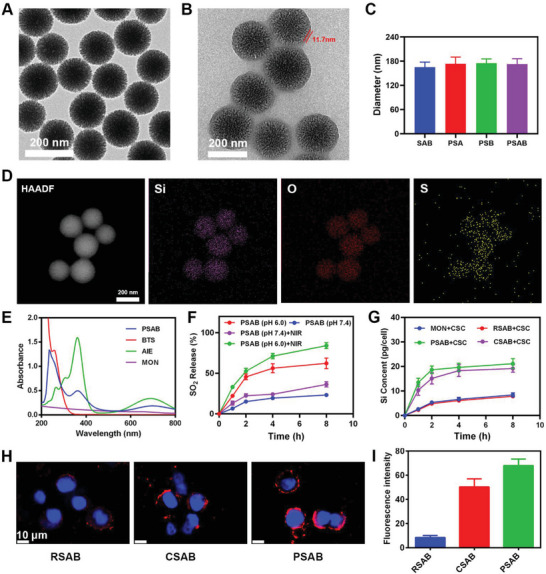
A) TEM image of MON. B) TEM image of PSAB. C) Hydrodynamic diameter of different materials. D) Elemental mapping of Si, O, and S in MON. HAADF: High‐angle annular dark field. E) Absorption spectra of MON, AIE, BTS, and PSAB. F) Fluorescence intensities of the DEACA probe after incubation with PSA or PSAB in different pH values for different periods of time. G) Nanoparticles are taken by CSCs after various incubation periods. H) CLSM images of 4T1 CSCs incubated with different nanoparticles for 2 h. DAPI: blue, Dil: red. Scale bars: 20 µm. I) Quantitative analysis of Dil fluorescence intensity in Figure 1H.

Currently, prevalent types of nanomaterials encompass carbon nanomaterials, MON, magnetic nanoparticles, and polymer nanomaterials.^[^ ^16^ ^]^ MON possesses several advantageous properties, including a large surface area, pore size adjustability, a well‐organized pore structure, high biocompatibility, and ease of surface modification.^[^ ^17^ ^]^ These properties make MON a suitable material for the construction of anticancer drug delivery platforms in multimodal therapy.^[^ ^16i^ ^]^ In this work, we fabricated the MON with disulfide bonds, which could be used in GSH‐responsive drug delivery research.^[^ ^10^ ^]^ Then the AIEgen named 2TT‐*o*C26B was prepared and characterized based on our previous reports (Figures S1–S3, Supporting Information),^[^ ^8c^ ^]^ which exhibits significant AIE properties (Figure S4, Supporting Information). 2TT‐*o*C26B has good photothermal cycling performance and high photothermal conversion efficiency (Figure S5, Supporting Information). Transmission electron microscopy (TEM) revealed that the prepared MONs had a size of ≈160 nm (**Figure** **1**A). As shown in Figure S6 (Supporting Information), MON can be degraded under GSH conditions. PSAB was synthesized utilizing a liposome extruder, and the morphology was characterized by the TEM image (Figure 1B). The monodisperse PSAB had a hydrodynamic diameter of 174.2 ± 5.4 nm (Figure 1C; Figure S7, Supporting Information) and a zeta potential of −16.1 ± 0.9 mV (Figure S8, Supporting Information). The major elements Si, O, and S were well located in the porous structure of MON (Figure 1D). In addition, PSA and PSB were fabricated as the control groups, which encapsulated only 2TT‐*o*C26B or BTS in MONs with coating by platelet membrane, respectively. The UV/vis–NIR absorption spectra simultaneously revealed the absorption bands of AIE‐active 2TT‐*o*C26B at 380 nm and BTS at 220 nm (Figure 1E), suggesting that 2TT‐*o*C26B and BTS were successfully encapsulated within PSAB (Figure S9, Supporting Information). The western blot results showed the presence of P‐selectin protein on the surface of PSAB. 7‐diethylaminocoumarin‐3‐aldehyde (DEACA), which reacts with bisulfite anion to produce blue fluorescence at 483 nm, was employed to validate the SO_2_ release.^[^ ^15b^ ^]^ As shown in Figure 1F, the release of SO_2_ from PSAB at pH 6.0 gradually increases over time and reaches up to 65% in 8 h, which is much higher than that from PSAB at PH 7.4 (22%), indicating that PSAB can continuously release SO_2_ under acidic conditions. As the control, PSA did not show any SO_2_ release due to the absence of SO_2_ prodrug, BTS. Subsequently, inductively coupled plasma‐atomic emission spectrometry (ICP‐AES) was utilized to examine the CSCs phagocytosis of various substances. Erythrocyte membrane encapsulated SAB nanoparticles, RSAB, were prepared as the control group. After treating CSCs with MON and RSAB for 8 h, respectively, the silicon concentrations of the two groups of cells were both ≈7.80 pg. In comparison, the silicon content was increased to ≈21.12 pg when CSCs were treated with PSAB (Figure 1G). Then, the internalization efficiency of CSCs for PSAB was verified by laser confocal scanning microscopy (CLSM) analysis. 4T1 cells were divided into two groups and incubated with Dil‐labeled PSAB or RSAB respectively. After two hours, the fluorescence intensity of Dil in the PSAB group was significantly stronger than that in the RSAB group, which may be because the platelet membrane endowed PSAB with powerful CSCs targeting ability (Figure 1H,I). The photothermal properties were further verified. As shown in Figure S10 (Supporting Information), the temperature of PSAB rose rapidly within 5 min upon 808 nm laser irradiation with concentration dependence.

### 2.2 In Vitro Antitumor Experiments

**Figure 2:**
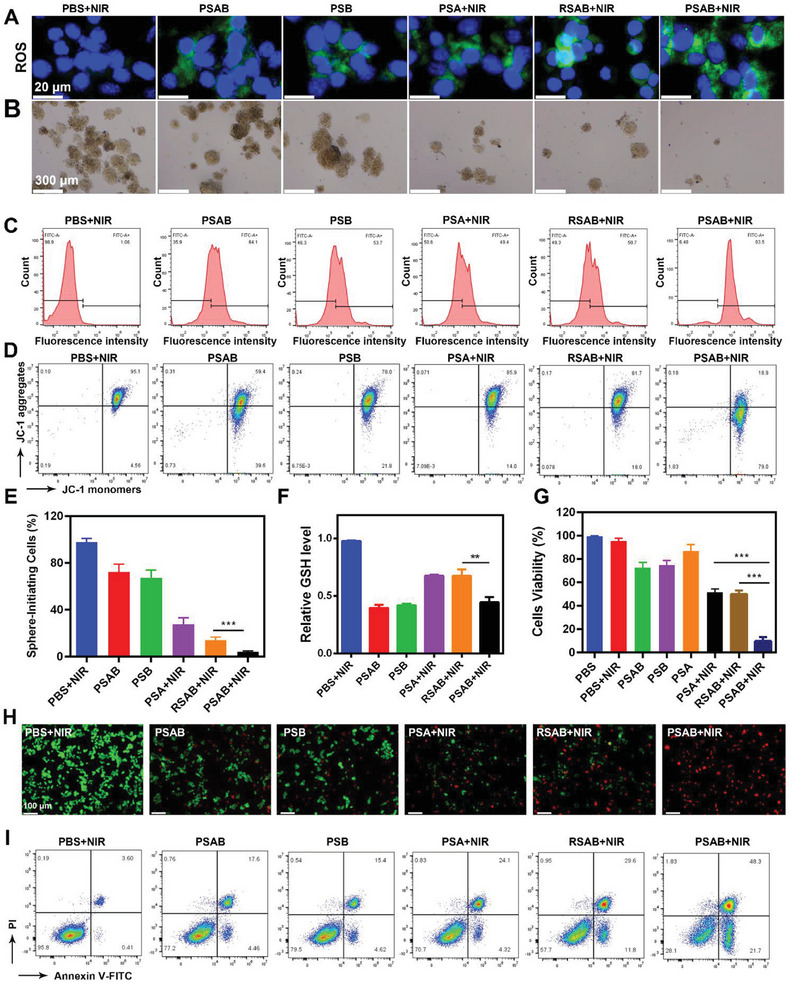
A) Fluorescence microscopy images of cancer cells for ROS detection with a DCFH‐DA probe. B) Sphere‐formation assays using 4T1 CSCs cultured with various treatments. C) Flow cytometric ROS analysis of 4T1 cells treated with various groups. D) Flow cytometric JC‐1 analysis of 4T1 cells treated with various groups. E) The numbers of tumor spheres were counted after various treatments. F) Reduction of GSH in cancer cells treated with different formulations. G) Cell viability of cancer cells after the indicated treatments. Statistical significance was calculated via one‐way ANOVA with Tukey's test: ^**^ *p* < 0.01, ^***^ *p* < 0.001.

Considering the excellent properties of prepared PSAB, we then explored the in vitro antitumor effect of PSAB on 4T1 CSCs. Studies have shown that CSCs can develop resistance to conventional cancer treatments by preventing oxidative stress.^[^ ^16^, ^18^ ^]^ The commercially available probe 2′,7′‐dichlorofluorescin diacetate (DCFH‐DA) was used to evaluate intracellular ROS production. As shown in **Figure** **2**A,C, the tumor cells treated with PBS+NIR exhibited no clear fluorescence, while limited green fluorescence signals were displayed after cells treated with the PSA+NIR group, PSAB group, PSB, and RSAB+NIR treatments. Elevated levels of ROS intensity were detected in 4T1 cells after treatment with PSAB+NIR. We subsequently evaluated the production of intracellular SO_2_, which plays a crucial role in depleting GSH in CSCs. As shown in Figure S11 (Supporting Information), the intracellular SO_2_ production in the PSAB, PSB, and PSAB+NIR groups was higher than that in the RSAB group. This may be due to the better tumor‐targeting ability of the platelet membrane, leading to more BTS accumulation. We further evaluated the effect of PSAB on CSCs proliferation by sphere‐formation assays. As shown in Figure 2B,E, the spheroid formation rate was reduced to 10% after treatment with PSAB+NIR groups with few spheroids formation, showing the most significant inhibition effect on CSCs spheroidization compared to other treatments. Next, the mechanism of the in vitro killing effect of PSAB+NIR on 4T1 CSCs was deciphered. Oxidative damage of cells by ROS is often accompanied by a decrease of mitochondrial membrane potential (MMP), which could be measured by the JC‐1 (5,5′,6,6′‐tetrachloro‐1,1′,3,3′‐tetraethyl‐imidacarbocyanine) probe.^[^ ^19^ ^]^ In a normal condition, JC‐1 dye would accumulate in the mitochondria and exhibit red fluorescence. When mitochondria are damaged and the MMP is decreased, the JC monomer is released into the cytoplasm, demonstrating green fluorescence.^[^ ^19^ ^]^ As shown in Figure 2D and Figure S12 (Supporting Information), the highest green/red ratio of JC‐1 fluorescent dyes in the PSAB+NIR group indicated the greatest mitochondrial depolarization. GSH remained at a high level in the “PSA + NIR” group. When treated with PSAB that contains BTS and exposed to NIR laser irradiation, the intracellular GSH levels were reduced by ≈60% in comparison to the control group, confirming the inhibitory effect of SO_2_ production on GSH (Figure 2F). This may be due to the loaded BTS could produce intracellular SO_2_, therefore reducing the GSH levels in CSCs. On the other hand, the disulfide bonds in MON could be broken down by GSH in the tumor cells, thereby facilitating the release of BTS and consuming GSH itself. According to the CCK‐8 cell viability test, the PSAB+NIR treatment had the highest CSCs inhibition compared with the other treatment (Figure 2G). PSAB has less toxicity to normal cells (Figure S13, Supporting Information). In addition, the staining experiments of live and dead cells and apoptosis experiments further demonstrated the killing effect of PSAB on 4T1 cells, as shown in Figures 2H,I. These discoveries implied that the combination of AIEgen‐meditated PTT and SO_2_ gas therapy in the CSCs‐targeting PSAB could effectively inhibit CSCs proliferation.

### 2.3 In Vivo Tumor Targeting and Biodistribution

**Figure 3:**
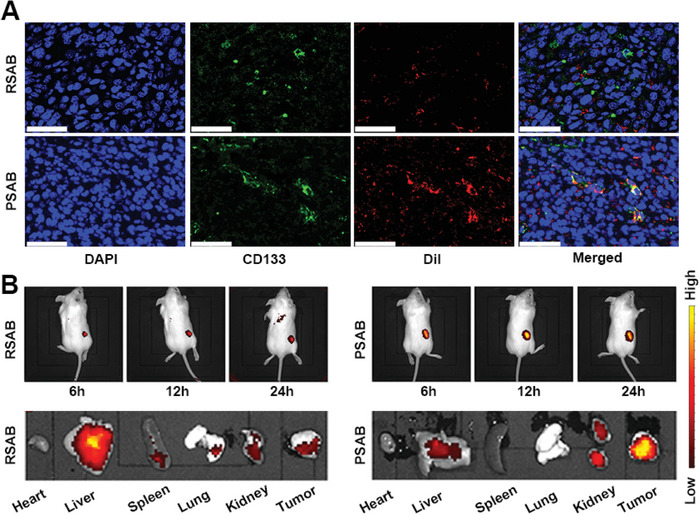
A) Colocalization of Dil (red), CD133 antibody‐labeled cancer cells (green), and nucleus (blue) in tumor sections of 4T1 tumor‐bearing mice 12 h after intravenous administration of indicated nanoparticles. Scale bars: 50 µm. B) Biodistribution profile of PSAB in *vivo* at different time points after injection and visceral fluorescence images after dissection at 24 h. *E*_x_ = 675 nm, *E*_m_ = 850 nm. Statistical significance was calculated via one‐way ANOVA with Tukey's test: ^***^ *p* < 0.001.

Considering the effective in vitro targeting effect of fabricated PASB, its in vivo behavior was further investigated. As shown in **Figure** **3**A, there was a substantial accumulation of Dil‐labeled PSAB in close proximity to the CD133‐expressed cancer cells. The biodistribution profiles of PSAB and RSAB were subsequently examined (Figure 3B). After a 12‐h treatment period, the majority of the administered nanoparticles predominantly accumulated in the liver of the mice in the RSAB group. Conversely, the PSAB group exhibited effective tumor targeting and less accumulation in main organs. This finding provides additional evidence reinforcing the targeted capabilities of the PSAB.

### 2.4 In Vivo Antitumor Effect

**Figure 4:**
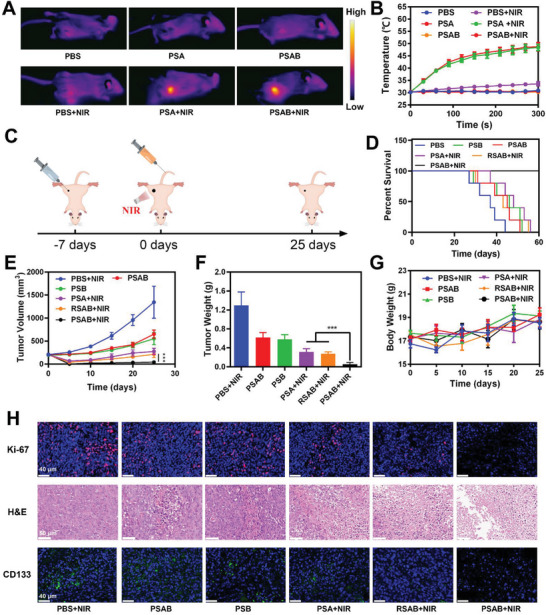
A) Infrared images of the mice under laser irradiation for 5 min after the indicated treatments. B) Temperature change of the tumor site upon laser irradiation. C) Schematic overview of the experimental model and treatment approach. D) Survival curves of tumor‐bearing mice after different treatments. E) Evolution of the volume of 4T1 tumors following different treatments. Data are displayed as the mean ± SD (*n* = 5). F) Evolution of the tumor weight after therapy. Data are displayed as the mean ± SD (*n* = 5). G) Body weight of nude mice recorded on alternate days for various treatments. Data are displayed as the mean ± SD (*n* = 5). H) Ki‐67, H&E, and CD133 stained tumor sections from the indicated treatment groups. Statistical significance was calculated via one‐way ANOVA with Tukey's test: ^***^ *p* < 0.001.

Infrared (IR) thermogram showed a negligible change of temperature in the PBS+NIR group (less than 2 °C increase), while the temperature of the tumor area increased to 45.2 °C after being treated by PSAB+NIR (**Figure** **4**A,B). This indicates that PSAB has a good in vivo photothermal effect.

**Figure 5:**
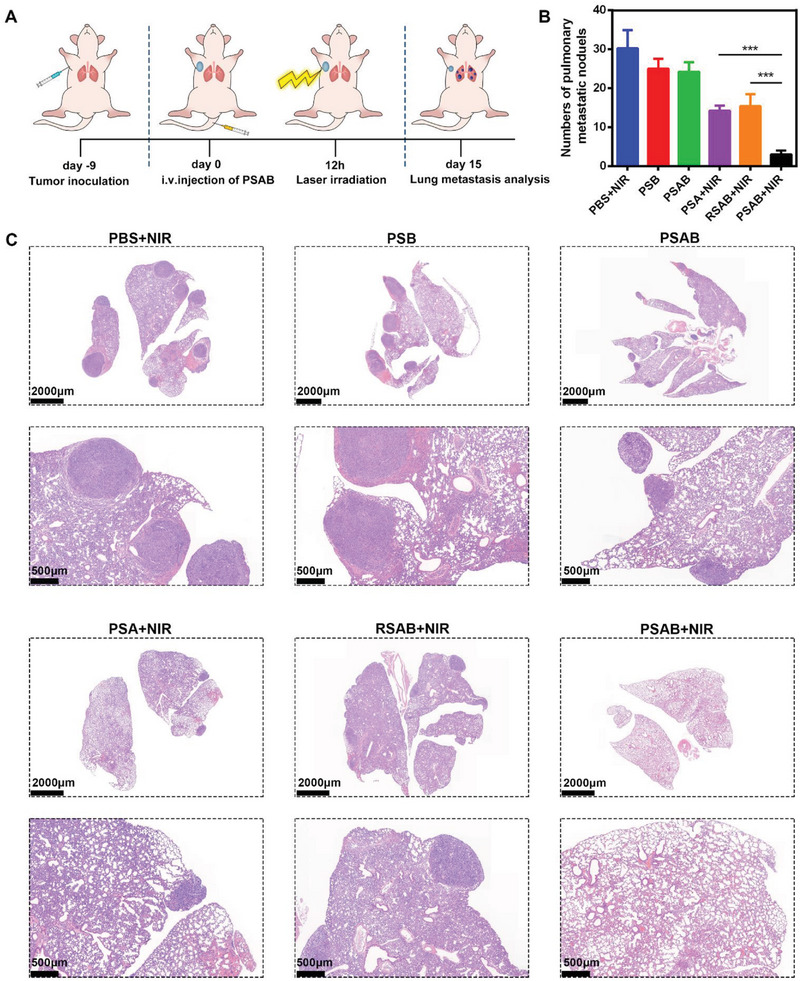
A) Schematic overview of the experimental model and treatment approach. B) Quantitative analysis of pulmonary metastatic nodules for every group. Data are displayed as the mean ± SD (*n* = 5). C) H&E staining of the lung tissues from the mice after indicated treatment with different magnifications. Statistical significance was calculated via one‐way ANOVA with Tukey's test: ^***^ *p* < 0.001.

Subsequently, the favorable in vitro anticancer properties and in vivo tumor targeting power of PSAB encouraged further exploration of its potential in vivo antitumor efficacy. A subcutaneous tumor model was established using the 4T1 cell line, as depicted in Figure 4C. When tumors reached 200 mm^3^, mice were randomly assigned to 6 groups: 1) PBS+NIR (808 nm, 0.5 W cm^−2^, 10 min); 2) PSAB; 3) PSB; 4) PSA + NIR; 5) RSAB + NIR; 6) PSAB + NIR. Compared to the PBS+NIR group, the survival period of PSB, PSAB, PSA+NIR, and RSAB+NIR treated mice have a longer survival time. Surprisingly, the PSAB + NIR treated mice all survived for >60 days and had the longest survival time (Figure 4D). Tumor volume exhibited a significant increase for the mice in the control groups (PBS + NIR, PSAB, and PSB). Although PSA + NIR and RSAB + NIR treatments induced initial tumor regression and slowed down subsequent tumor regrowth in the following days, tumor suppression was not complete and tumor recurrence still occurred. In contrast, the tumor in the PSAB + NIR group was controlled throughout the treatment period (Figure 4E). Notably, the PSAB + NIR group demonstrated a significant reduction in tumor weight, with an average weight of only 0.08 g (Figure 4F; Figure S14, Supporting Information). This finding suggests that the combined therapeutic approach had the most pronounced effect in inhibiting tumor growth and recurrence. Furthermore, no significant changes in the body weights of mice across all groups were observed (Figure 4G), suggesting the absence of apparent adverse effects associated with this treatment approach. Furthermore, the PSAB + NIR group exhibited the most extensive tumor necrosis, growth suppression, and CSCs elimination, as evidenced by H&E, Ki‐67, and CD133 immunofluorescence staining of tumor tissue sections (Figure 4H). We further evaluated its effect on the metastasis inhibition of the mice (**Figure** **5**A). As shown in Figure 5B,C, the lung metastases in mice treated with PSAB+NIR were significantly reduced, resulting in the lowest incidence of lung metastasis. Previous studies have highlighted the critical role of CSCs in tumor metastasis.^[^ ^20^ ^]^ Therefore, the PSAB system, confirming efficient CSCs ablation power with the combination of gas therapy and PTT, also demonstrated the ability to inhibit tumor metastasis.

**Figure 6:**
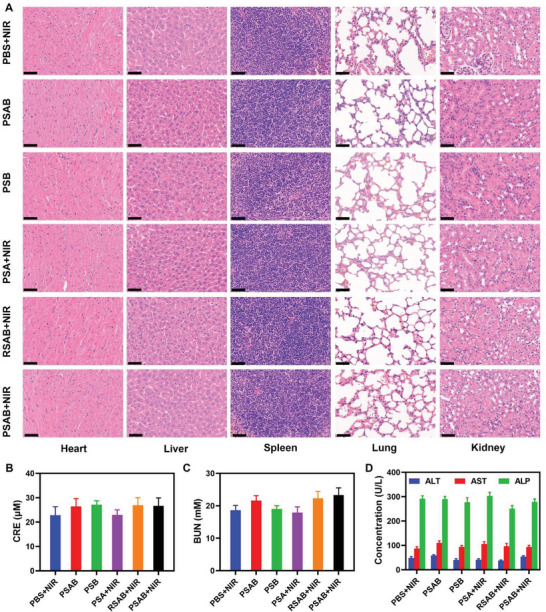
A) Histopathological analysis results (H&E‐stained images) of the major organs of mice that were exposed to different treatments. Scale bars: 40 µm. B) The kidney function markers: CRE and C) BUN from mice after different treatments. Data are displayed as the mean ± SD (*n* = 5). D) The liver function markers: ALT, ALP, and AST from mice after different treatments. Data are displayed as the mean ± SD (*n* = 5).

After the treatment, the mice were sacrificed and the major organs (liver, heart, spleen, kidney, and lung) were taken for histological analysis. Notably, tissue slices from various organs showed no signs of inflammation or injury. There were no indications of liver or kidney dysfunction from blood biochemistry evaluation, indicating the higher biological safety of PSAB (**Figure** **6**). These findings underscore the favorable characteristics of PSAB in terms of biosafety.

## 3 Conclusion

In conclusion, PSAB was designed as a novel drug delivery system to prevent insufficient PTT‐induced tumor recurrence and metastasis. PSAB could mainly target CSCs in tumor tissue after intravenous injection. Overexpressed GSH in the CSCs microenvironment facilitated the intracellular release of SO_2_ gas, which reversely depleted GSH due to the oxidative power of SO_2_ and enhanced AIEgen‐mediated PTT effect. Thus, the combination of gas therapy and AIEgen‐mediated PTT in PSAB can effectively eliminate CSCs and inhibit recurrence and metastasis. This research describes the advancement of a highly accurate CSCs elimination strategy, which holds potential as an innovative therapeutic strategy for various forms of cancer. The findings of the current study have the potential to inform the development of innovative nano‐technologies for incomplete PTT.

## Conflict of Interest

The authors declare no conflict of interest.

## Supporting information

Supporting Information

## Data Availability

The data that support the findings of this study are available from the corresponding author upon reasonable request.
