# Supplementary material for: Biomimetic Nanosystem Loading Aggregation‐Induced Emission Luminogens and SO2 Prodrug for Inhibiting Insufficient Photothermal Therapy‐Induced Breast Cancer Recurrence and Metastasis
Source: Adv Sci (Weinh). 2024 Jul 21;11(35):2405575. doi: 10.1002/advs.202405575 (PMC11425245; doi:10.1002/advs.202405575)
Supplement: Supplementary file 1 — Supporting Information [file ADVS-11-2405575-s001.docx]

Experimental Procedures

**Materials and reagents.**

Tetraethoxysilane (TEOS), hexadecyl trimethyl ammonium bromide (CTAB), bis[3-(triethoxysilyl)propyl] tetrasulfide (BTES) and triethanolamine (TEA) were obtained from Sigma–Aldrich. 2′,7′-Dichlorofluorescin diacetate (DCFH-DA) and Dil (DiIC18(3)) were obtained from Solarbio life sciences. GSH Assay Kit was purchased from Beyotime Institute of Biotechnology (China). Sodium salicylate (NaSal) was obtained from Sinopharm Chemical Reagent Co., Ltd. All of the aqueous solutions were prepared using purified deionized (DI) water purified with a purification system (Direct-Q3, Millipore, USA). The other solvents used in this work were purchased from Sinopharm Chemical Reagent (China) and Aladdin-Reagent (China).

**Cell culture**

4T1 cell lines and RAW 264.7 cells were obtained from the Cell Bank of the Chinese Academy of Sciences and incubated in RPMI-1640 or DMEM medium supplemented with 10% FBS in a humidified atmosphere.

The cancer stem cells (CSC) were sorted from 4T1 cells and cultured as guided by the previous report^[^[^1^](#_ENREF_1)^]^. 4T1 cells suspension (10000 cells/ml) were seeded in ultra-low attachment surface 6-well plates (Corning, USA) with serum-free DMEM-F12 medium, containing B27 (1:50), epidermal growth factor, basic fibroblast growth factor (20 ng/mL), N2 additives. Primary cultures of colonospheres were harvested after 7 days in culture and collected after centrifugation, dissociated with Trypsin–EDTA. The expression of CD44 and CD133 on CSC were tested by flow cytometry.

The Non-cancer stem cells (nCSC) were also sorted from 4T1 cells. Briefly, 4T1 cells were split in Serum Containing Medium (SCM). This is made up of DMEM/Ham’s F12 (1:1) (Hyclone), 10 % Foetal Bovine Serum(FBS) (Biological Industries), 1 % penicillin/streptomycin (P/S) (100 U/ml) (Hyclone), and 1 % L-glutamine (LG) (Lonza). The adherent cells obtained from the SCM were harvested by trypsinization to obtain single cells and then expanded in SCM medium to obtain more cells. These cells were scored as non-cancer stem cells (nCSC).

**Preparation and characterization of MON**

MON were achieved via a one-pot synthesis referring to the work reported previously^[^[^2^](#_ENREF_2)^]^. First, 50 mL of water with 0.25 g of TEA was stirred gently in an oil bath under a magnetic stirring for 30 min at 80 °C, then 760 mg of CTAB and 168 mg of NaSal were added to the above solution and kept stirring for 30 min. After that, 4 mL of TEOS and 3.2 mL of BTES premixed solution were added to the above solution with robust stirring. After 12 h reaction, the products were collected by high-speed centrifugation and washed three times with ethanol. Then, the collected products were re-dispersed in 1% NaCl methanol solution at room temperature for 6 h for three times to remove the template. After that, the obtained MON were re-dispersed in deionized water for further use.

**Preparation and characterization of PSAB**

The platelet membrane vesicles (PV) and red blood cell membranes (RM) were prepared according to previous work^[^[^3^](#_ENREF_3)^]^. The 2TT-*o*C26B were prepared according to our previous work^[^[^4^](#_ENREF_5)^]^. MS: m/z: [M]+ calcd for C_66_H_64_N_6_S_4_: 1068.4075, found: 1068.4075.

2TT-*o*C26B (10 mg) was dissolved in 1 mL of DMSO, and then the solution was added to 8 mL PBS solution containing 0.5 mg of MON and 1mg BTS under gentle stirring for 6h. Next, the solution was centrifuged at 7000 rpm for 10 min to obtain the AIE and BTS co-loaded MON (SAB). Finally, the SAB was mixed with 1mg PV and then repeatedly coextruded through 200 nm pores. The resultant PSAB particles were centrifuged and washed with PBS several times to remove the excess PV. Drug loading capacity was calculated by UV-vis spectra at the UV-vis spectrophotometry Lambda 35 (Perkin-Elmer). Loading capacity = M_drug_/M_PSAB_. where M refers to the mass. Red blood cell membrane biomimetic nanoplatform (RSAB) was prepared in the same way as PSAB. The zeta potential and diameter of nanoparticles were measured by dynamic light scattering (DLS, Nano-Zen 3600, Malvern Instruments, UK). The morphological structures of different formulations and the elemental mapping images were captured by JEOL JEM-2100F TEM.

***In vitro* cancer targeting study**

4T1 CSC were seeded in 24-well plates and cultured for 12 h. Then RSAB and PSAB were added to the medium (100ug/mL MON). Then the cells were washed three times and incubated for 4 h at 37 °C, 5% CO^2^, and then washed with PBS three times. The cells were then fixed with PFA for 30 min at room temperature, stained with DAPI, and then imaged by using a confocal laser scanning microscope (CLSM; IX81, Olympus, Japan). The fluorescence intensity was measured by a flow cytometer.

4T1 CSC were seeded in 12-well plates and cultured for 12 h. Then the Si, RSAB, and PSAB were added to the medium (100ug/mL MON). Then the cells were washed three times and incubated for 4 h at 37 °C, 5% CO_2_, and then washed with PBS three times. To quantify Si uptake, the cells were lysed by adding 0.5 mL 1% Tween 80 to each well. The cell lysate from each well was then added to 10 mL HCl and heating to dissolve. Boil the mixture and cool to room temperature. The sample was then resuspended with 100 mL DI water and the Si content in each sample was determined with ICP-AES.

**Photothermal Conversion Efficiency**

An 808 nm NIR laser (Changchun New Industries Tech.Co., Ltd., Changchun, China) with irradiation powers was used. The photothermal images of the PSAB and 2TT-*o*C26B during laser irradiation were recorded every 30 s using an infrared thermal imaging system. The NIR laser source was equipped with a 4 mm diameter laser module with an adjustable power. The photothermal conversion efficiency was calculated using the following equation^[^[^5^](#_ENREF_6)^]^:


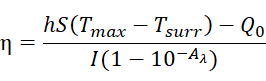


where h is the heat transfer coefficient, S is the surface of the container, T_max_ and T_surr_ are the equilibrium temperature and ambient temperature, respectively. Q_0_ is the heat associated with the light absorbance of the solvent, A_λ_ is the absorbance of 2TT-*o*C26B at 808 nm, and I is the laser power density. According to the above equation, the η value of 2TT-*o*C26B was determined to be about 39.1%.

**Intracellular ROS and SO_2_ detection**

4T1 CSC (8 × 10^4^ per plate) were incubated with six different groups: (1) PBS+NIR (808nm, 0.5W/cm^2^, 10min); (2) PSAB; (3) PSB; (4) PSA + NIR; (5) RSAB + NIR; (6) PSAB + NIR. The BTS concentration was 50μg/mL. Then, the fluorescent dye, DCFH-DA (10 μM) or 7-Diethylaminocoumarin-3-aldehyde (DEACA, 10 μM), was added and co-incubated for 10 min at 37 °C. Then, cells in groups 1, 4, 5, and 6 were exposed to NIR irradiation for 10 min and were detected by a confocal laser scanning microscope (CLSM; IX81, Olympus, Japan).

**JC-1 detection *in vitro***

4T1 CSC (8 × 10^4^ per plate) were incubated with six different groups: (1) PBS+NIR (808nm, 0.5W/cm^2^, 10min); (2) PSAB; (3) PSB; (4) PSA + NIR; (5) RSAB + NIR; (6) PSAB + NIR. The LAP concentration was 2μg/mL in groups 2 and 5. Then, the fluorescent dye, JC-1 (10 μM) was added and co-incubated for 10 min at 37 °C. Then, cells in groups 2, 3, 5, and 6 were exposed to white light radiation for 10 min and was detected by a confocal laser scanning microscope (CLSM; IX81, Olympus, Japan) and Flow cytometry.

**Detection of Intracellular GSH**

The commercially available GSH assay kit was used to detect the depletion of GSH. 4T1 CSC (8 × 10^4^ per plate) were incubated with six different groups: (1) PBS+NIR (808nm, 0.5W/cm^2^, 10min); (2) PSAB; (3) PSB; (4) PSA + NIR; (5) RSAB + NIR; (6) PSAB + NIR. The BTS concentration was 50μg/mL. Then, cells in groups 1, 4, 5, and 6 were exposed to 808 nm laser radiation (0.5 W/cm^2^) for 5 min, each culture bottles were rinsed with PBS and centrifuged at 3000 rpm to collect cells. Then, the cells were suspended in 1 mL of PBS and processed by an ultrasound cell crusher. After that, 0.5 mL of the above cells was added to 2 mL of regent one in an assay kit and centrifuged at 3500 rpm for 10 min. The depletion of intracellular GSH was measured by UV−vis spectroscopy.

**Cytotoxicity in CSC‑enriched 3D tumorsphere cells**

Tumorspheres that were developed from 5000 CSC in 24-well ultralow attachment plates were treated with (1) PBS+NIR (808nm, 0.5W/cm^2^, 10min); (2) PSAB; (3) PSB; (4) PSA + NIR; (5) RSAB + NIR; (6) PSAB + NIR. The BTS concentration was 50μg/mL. Then, cells in groups 1, 4,5, and 6 were exposed to 808 nm laser radiation (0.5 W/cm^2^) for 5 min. After 5 days of incubation, take pictures of the tumorsphere and count the number.

**Cell viability and apoptosis assays**

Cell viability assays were conducted using a Cell Counting Kit-8 (CCK-8, Beyotime, China). CSCs were incubated in 96-well plates (5 × 10^4^ cells/well). The next day, the cells were treated with 8 different groups: (1) PBS+NIR (808nm, 0.5W/cm^2^, 10min); (2) PSAB; (3) PSB; (4) PSA + NIR; (5) RSAB + NIR; (6) PSAB + NIR; (7) PBS; (8) PSA. The BTS concentration was 50μg/mL. Then, cells in groups 1, 4, 5, and 6 were exposed to 808 nm laser radiation (0.5 W/cm^2^) for 5 min. After incubation, cell viability was determined by CCK-8 according to the instructions. To investigate the effect of PSAB on the activity of normal cells, we conducted similar experiments on RAW264.7 cells.

Cellular apoptosis was assessed by plating 4T1 cells in 6-well plates and treating them via the five methods listed above for 12 h. After an additional 12 h, cells were harvested with EDTA-free trypsin, and annexin V-FITC/PI (Beyotime Biotechnology Co., Shanghai, China) was used to analyze cellular apoptosis via flow cytometry.

**Animal tumor models**

Female Balb/c mice aged 4-5 weeks were purchased from Vital River Company (Beijing, China). 100 μL of 4T1 cell suspension (2×10^5^ cells) were subcutaneously injected into each mouse to establish the tumor models. The animal experiments were carried out according to the protocol approved by the Ministry of Health in People’s Republic of PR China and were approved by the Experimental Animal Welfare Ethics Committee of Guangxi Medical University.

***In vivo* bio-distribution study**

The 4T1 tumor model was used. When tumors reached 300mm^3^, tumor bearing mice (n = 3) received an intravenous (i.v.) of 100 μL PBS containing RSAB or PSAB (with a AIE dose of 20 mg/kg). Mice were anesthetized at different time points and subjected to in vivo imaging analysis using the IVIS system (Ex=675nm, Em=850nm). The tumor tissue and major organs of the mice were removed after experiments for tissue imaging analysis.

***In vivo* antitumor study.**

The 4T1 tumor model was used. When tumors reached 200mm^3^, tumor bearing mice were divided randomly into six different group (n=5): (1) PBS+NIR (808nm, 0.5W/cm^2^, 10min); (2) PSAB; (3) PSB; (4) PSA + NIR; (5) RSAB + NIR; (6) PSAB + NIR. The AIE dose was 20 mg/kg. Mice body weight and tumor volume in all groups were monitored every 5 days. A caliper was employed to measure the tumor length and tumor width and the tumor volume was calculated according to following formula. Tumor volume = tumor length × tumor width^2^ / 2. After 25 days treatment, mice were sacrificed. Tumors of all mice were harvested, washed with PBS, and fixed with paraformaldehyde for histology analysis. And the tumor tissues were weighed, and fixed in 4% neutral buffered formalin, processed routinely into paraffin, and sectioned at 4 μm. Then the sections were stained with Ki67, HE, and CD133 finally examined by using a confocal laser scanning microscope (CLSM; IX81, Olympus, Japan). At 25th day post the injection, the blood samples from these mice (≈1 mL) were collected for blood biochemistry analysis. The major organs including heart, liver, spleen, lung, and kidney were harvested, fixed in 4% of formalin, embedded in paraffin, sectioned into 4 μm slices, stained with hematoxylin and eosin (H&E), and observed by an optical microscope (BX51, Olympus, Japan).

***In Vivo* Antimetastasis Effect**

The in *vivo* orthotopic breast tumor model was established by subcutaneously injecting 9 × 10^5^ 4T1 cells into the right mammary gland of Balb/c nude mice. When the tumor volume grew to about 200 mm^3^ after injection, the mice were randomly assigned to 6 different groups (each group included 5 mice): (1) PBS+NIR (808nm, 0.5W/cm^2^, 10min); (2) PSAB; (3) PSB; (4) PSA + NIR; (5) RSAB + NIR; (6) PSAB + NIR. The AIE dose was 20 mg/kg. After 15 days, the mice were sacrificed, and the lungs of each group were collected. The metastatic nodules on the pulmonary tissues were counted, and the lung sections were stained by hematoxylin and eosin (H&E).

**Statistical analysis**

Data analyses were conducted using the GraphPad Prism 5.0 software. For variance analysis, One-way analysis of variance (ANOVA) with Tukey’s post hoc test was used. p values of <0.05 were considered significant. *p < 0.05, **p < 0.01, ***p < 0.001.

**Supplementary figures**


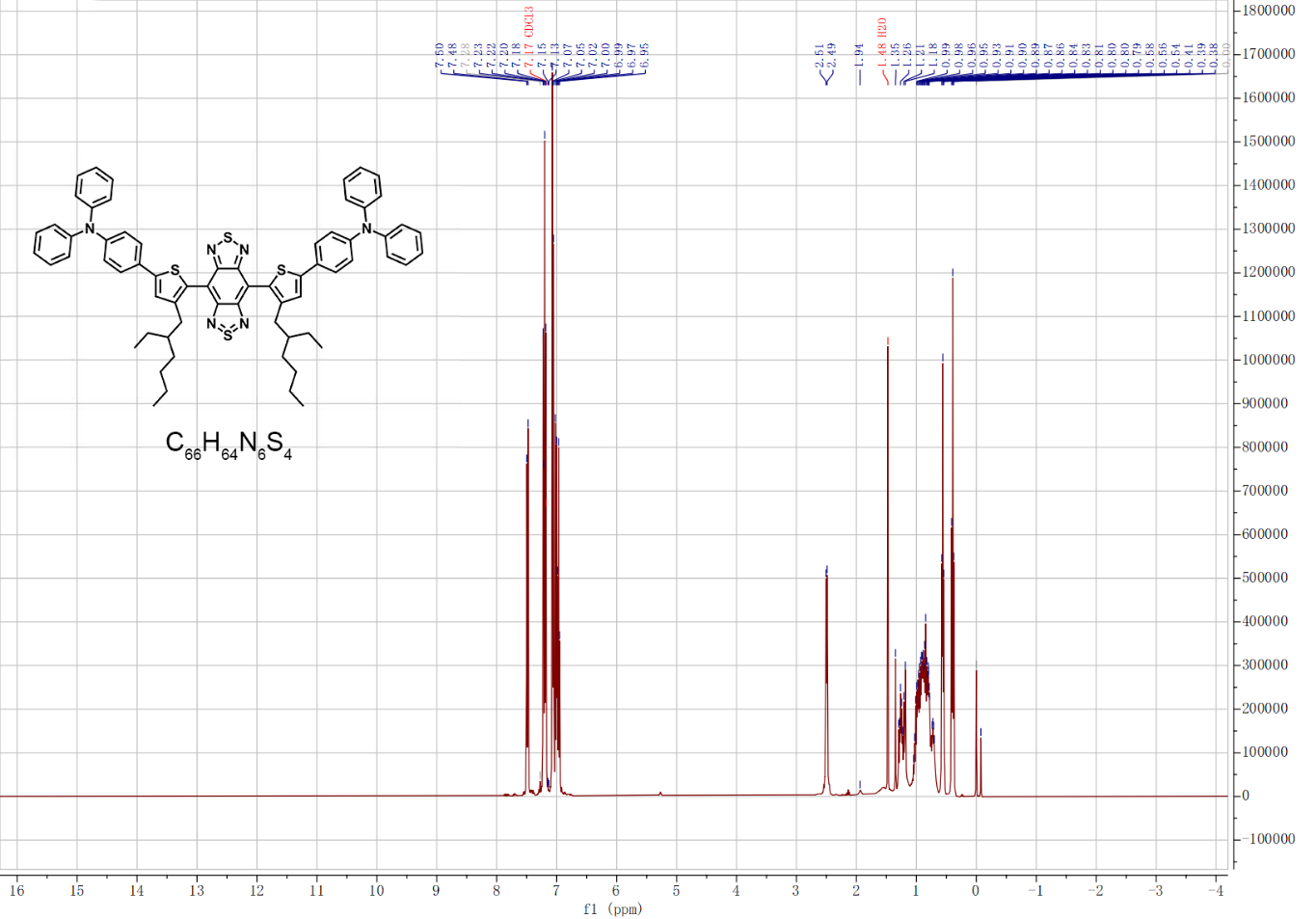


Figrue S1.^1^H NMR spectrum of 2TT-*o*C26B.


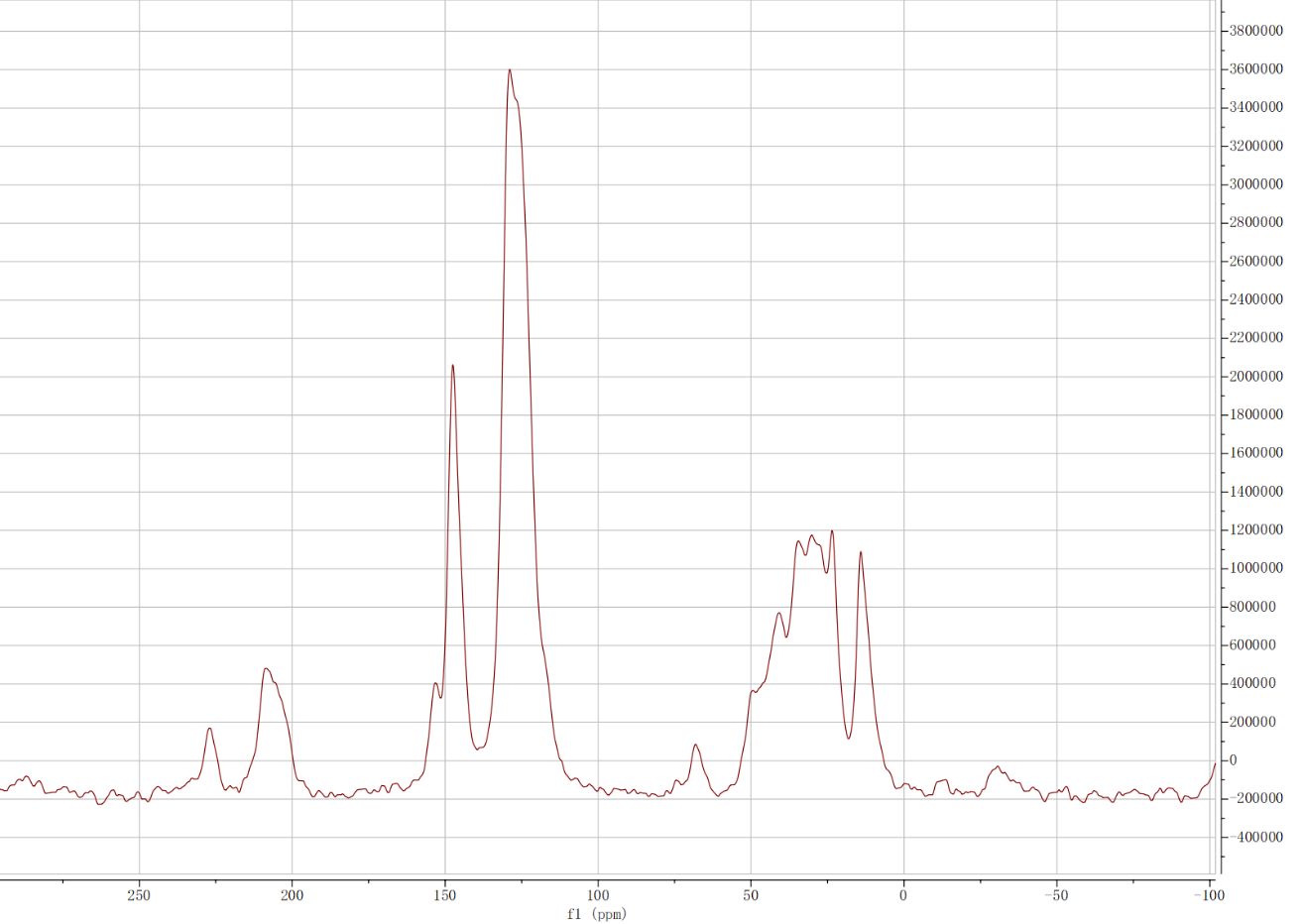


Figrue S2.^13^C Solid State NMR spectrum of 2TT-*o*C26B.


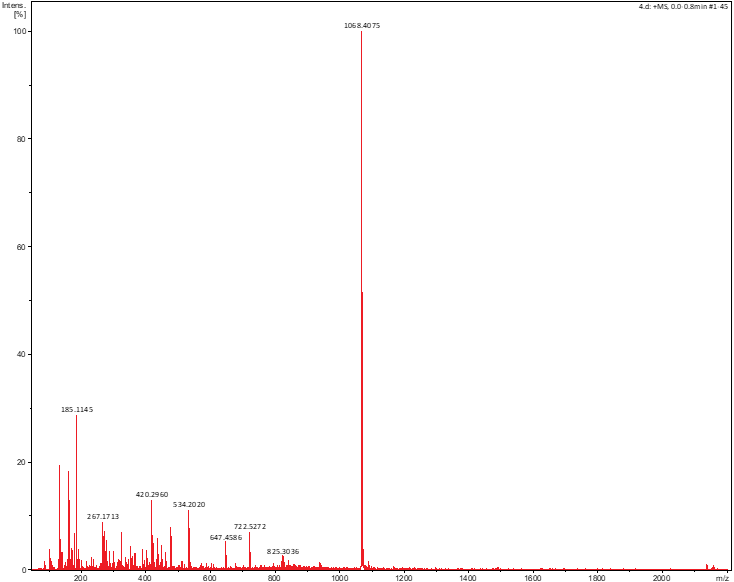


Figrue S3. High-Resolution Mass Spectrometry (HRMS) of 2TT-*o*C26B.


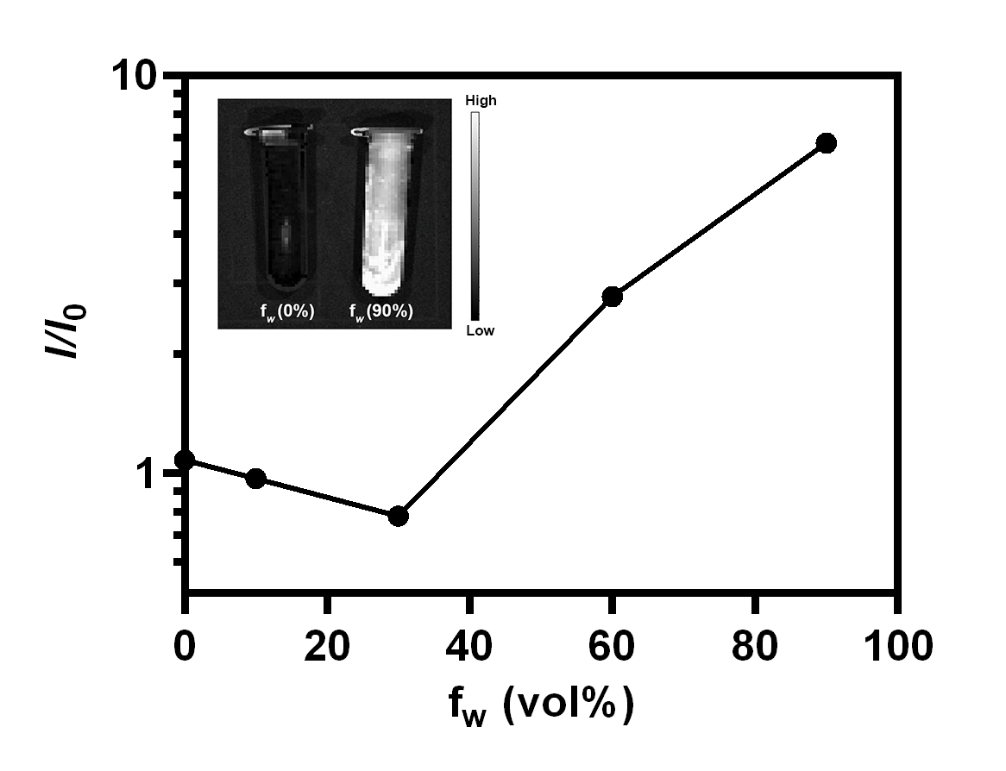


Figure S4. αAIE curves of 2TT-*o*C26B in THF/water mixtures with different water fraction (f_w_). Insert: Comparison of fluorescence signals under different f_w_. E_x_=675nm, E_m_=850nm.


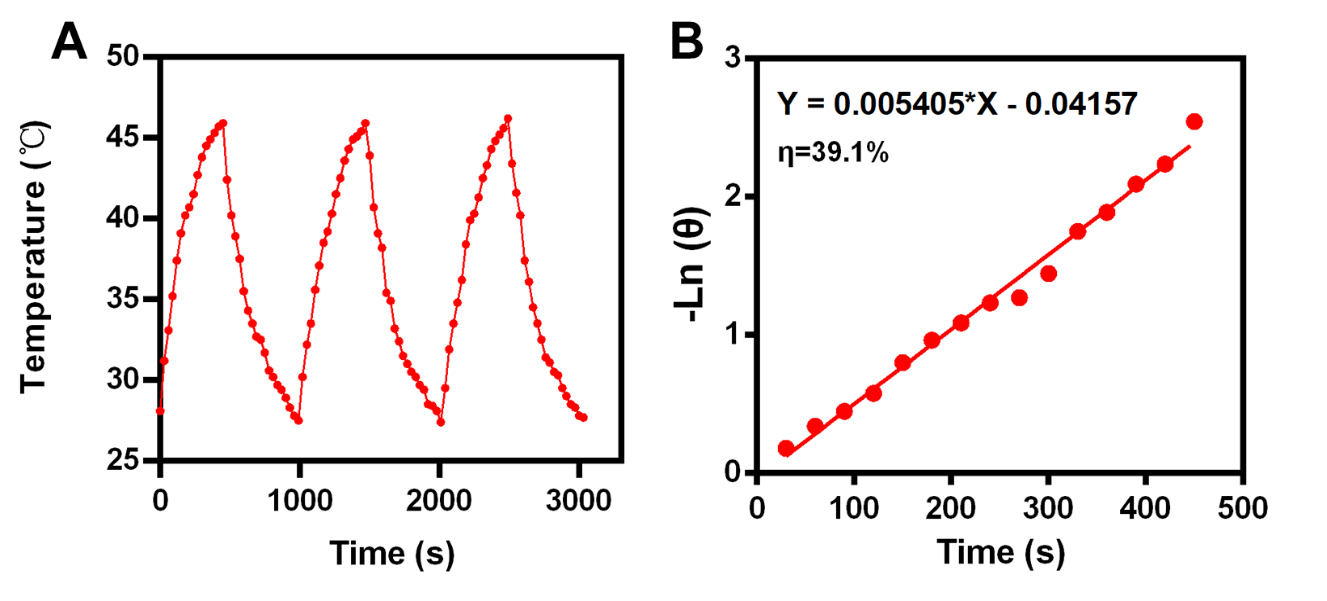


Figure S5. (A) Photothermal stability of 2TT-*o*C26B (200 μg/mL) during three warming-cooling cycles irradiated with 808 nm laser (1 W cm−2). (B) The photothermal conversion efficiency of 2TT-*o*C26B, plot of time versus −ln(𝜃) of 2TT-*o*C26B, where 𝜃 is the driving force for temperature.


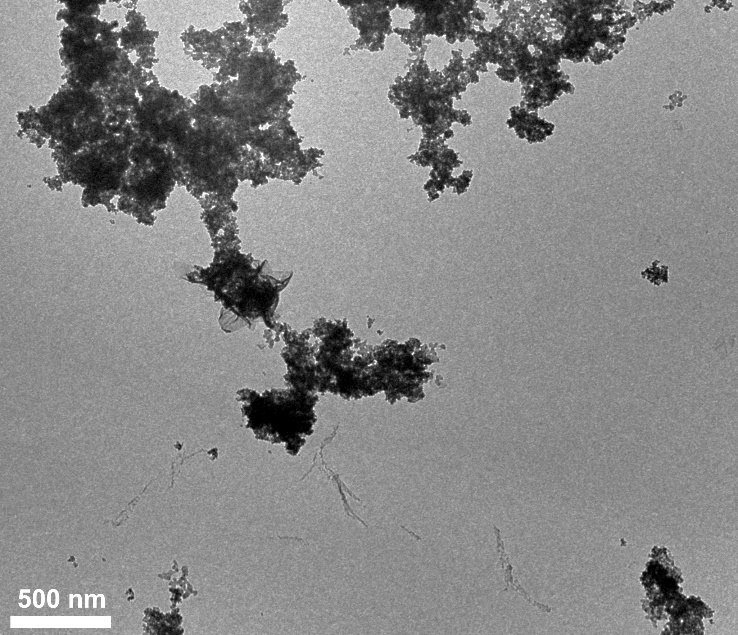


Figure S6. TEM images of MON after incubated with 10mM GSH for 12 h.





Figure S7. The particle size distribution of SAB and PSAB (n=3).





Figure S8. The zeta potential of SAB, PV and PSAB (n=3).


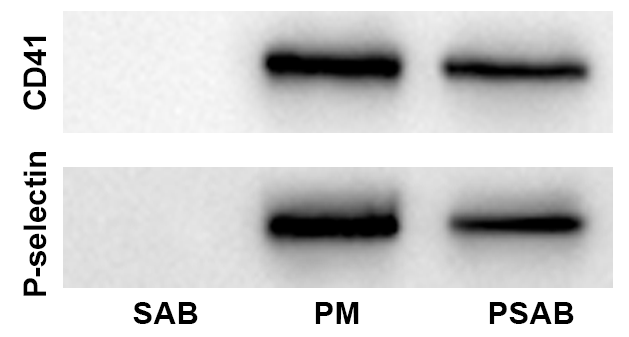


Figure S9. Platelet markers, including CD41 and P-selectin, were detected using western blotting.


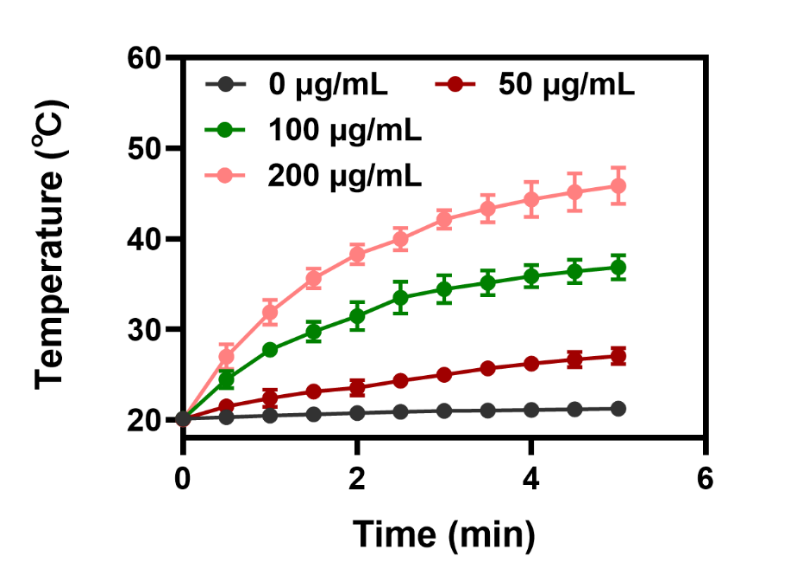


Figure S10. Temperature changes of PSAB at various AIE concentrations under 5 min irradiation from 808 nm laser at 0.5 W/cm^2^ (n=3).


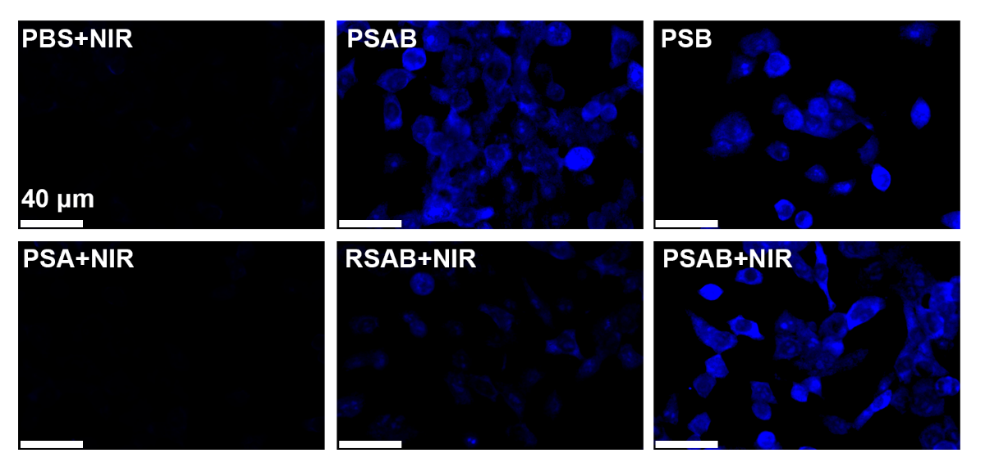


Figure S11. Detection of intracellular SO_2_ after different treatments.


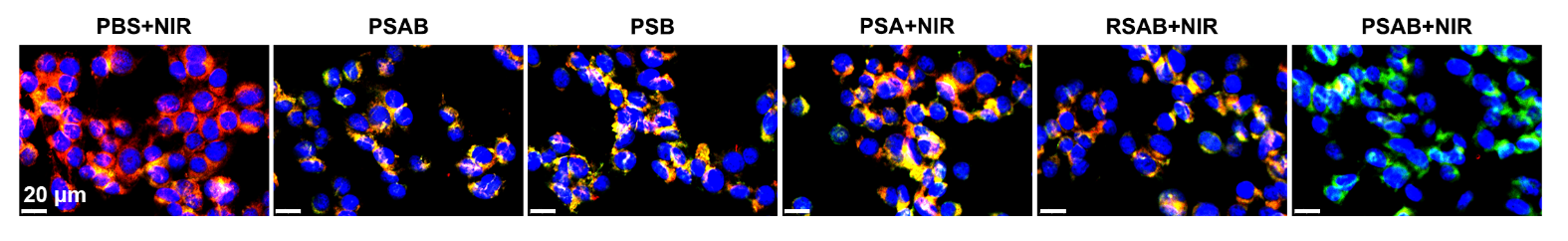


Figure S12. The fluorescence images of mitochondria dysfunction in cells detected by a mitochondria potential JC-1 probe (red fluorescence: normal mitochondria, green fluorescence: abnormal mitochondria).





Figure S13. Cell viability of RAW264.7 cells after the indicated treatments.


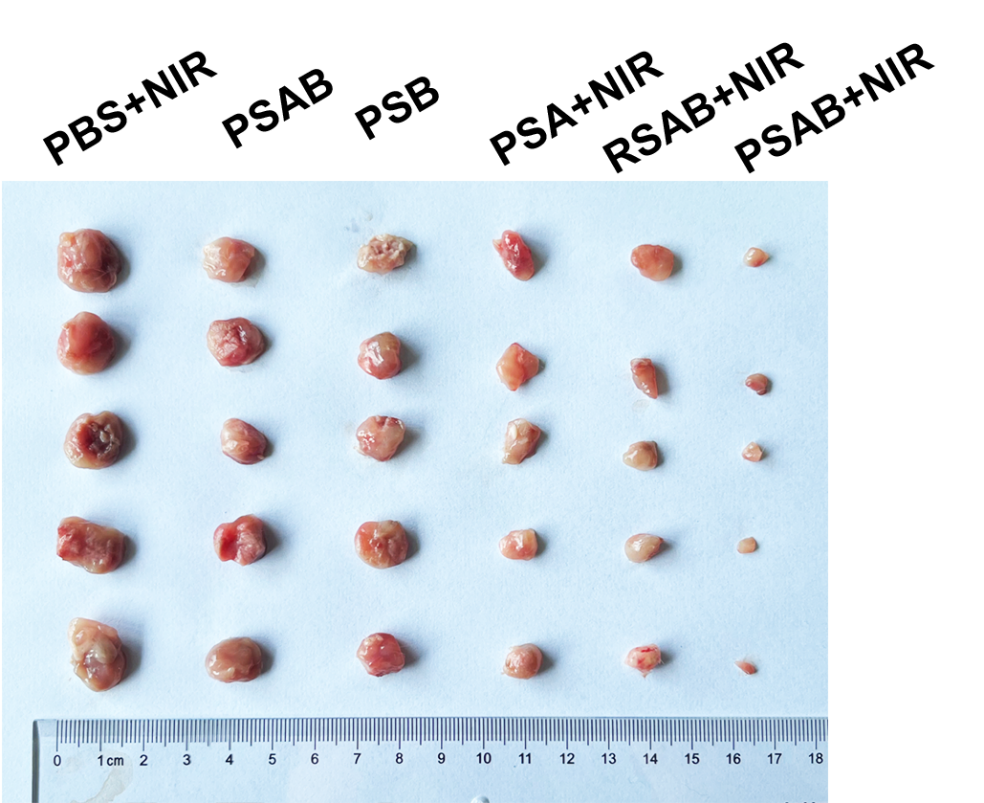


Figure S14. Representative digital photos of tumors collected from various groups.

**References**

[1] X. Xie, K. Jiang, B. Li, S. Hou, H. Tang, B. Shao, Y. Ping, Q. Zhang, *Biomaterials* **2022**, 121598.

[2] Y. Liu, S. Zhai, X. Jiang, Y. Liu, K. Wang, C. Wang, M. Zhang, X. Liu, W. Bu, *Advanced Functional Materials* **2021**, 31, 2010390.

[3] a)D. M. Zhu, W. Xie, Y. S. Xiao, M. Suo, M. H. Zan, Q. Q. Liao, X. J. Hu, L. B. Chen, B. Chen, W. T. Wu, L. W. Ji, H. M. Huang, S. S. Guo, X. Z. Zhao, Q. Y. Liu, W. Liu, *Nanotechnology* **2018**, 29, 084002; b)Y. Chen, G. Zhao, S. Wang, Y. He, S. Han, C. Du, S. Li, Z. Fan, C. Wang, J. Wang, *Biomater Sci* **2019**, 7, 3450.

[4] Y. Li, Z. Cai, S. Liu, H. Zhang, S. T. H. Wong, J. W. Y. Lam, R. T. K. Kwok, J. Qian, B. Z. Tang, *Nature communications* **2020**, 11, 1255.

[5] D. Zhu, M. Lyu, Q. Huang, M. Suo, Y. Liu, W. Jiang, Y. Duo, K. Fan, *ACS applied materials & interfaces* **2020**, 12, 36928.
